# Some Notes on Progress in Medicine

**Published:** 1907-11-16

**Authors:** 


### Some Notes on Progress in Medicine.

The commencement of the winter session's activities in the various professional societies, and in other more or less allied organisations, has recently afforded an opportunity for the delivery of a number of inaugural addresses, and some of these are to be recognised as of more than usual interest. As it happens, the speakers this year have included several prominent physicians, and thus it has come about that the position and prospects of what is termed "pure medicine" have been somewhat prominently discussed. The discussion, interesting in itself, is, however, by no means of importance merely to the physician. To a large extent, and possibly to an increasing extent, general practice has a distinctly medical, as opposed to a surgical quality, and the movement of medical knowledge and treatment is, therefore, an influence which concerns the fortunes of the great majority of the profession. There is a still larger circle which is affected by the progress of medical knowledge—namely, the general public. Hence the views and anticipations of experienced physicians in regard to the present position and future prospects of the art and science of medicine form a subject which commands the interest of a large and important constituency.

The physicians whose deliverances we have now in view recognise, perhaps with a certain air of sadness, that it is hopeless for them to claim for their branch of the healing art anything which can compete in the public judgment with the dramatic triumphs and achievements of the surgeon. Indeed, one of them tells us that of late medicine has become very surgical, and another, while not sparing in criticism of his surgical colleagues, speaks in terms of disparagement of the days when treatment consisted in "prescribing drugs and giving some directions about diet." Yet this tone is not one which marks all the utterances now under review. The President of the Medical Society, indeed, does not hesitate to claim that the advance of medicine has during the last thirty years been definitely greater than that of surgery, and that the future will far outstrip the past. To support this conclusion, the President indulged in a rapid but effective survey of the whole clinical field, and certainly he did not fail to produce some evidence for the faith which he professed. The period in question, it must be acknowledged, includes some of the most striking and impressive advances which the history of medicine can boast. Take, for example, the discovery by Koch of the bacillus of tubercle, and everything that has flowed from this; or the question of immunity, and the discovery and application of serum-therapeutics; or, once more, the organised movement of investigation and advance in various tropical diseases. Without question these are influences which have exercised a commanding position in the medicine of the past thirty years, and their range and force are by no means yet exhausted. It is of especial interest to observe how closely these and allied discoveries touch the practical affairs of life. There seems no question that, for example, without the modern knowledge of yellow fever the present attempt to construct the Panama Canal would have failed as disastrously as previous efforts. The first command was to "make the dirt fly," but it was found that until the physicians and bacteriologists had got rid of yellow fever, labourers could not be kept alive to carry out this energetic injunction. In face of this it is no idle dream that when, by the aid of medical science, yellow fever and malaria have been banished from the tropics, these regions may become habitable by the white races, and the entire face and history of the world be profoundly modified. And, to come nearer home, in view of what, during the last quarter of a century, has happened in the case of typhus fever, is it not possible that the next generation may see the practical suppression of tuberculosis? Certainly a profession which has offered these various practical contributions to the "relief of man's estate" has a right to protest its value and importance.

The suggestions which arise from this rapid survey seem to be two in number. First, it may be anticipated that surgery will continue to extend the range of its activities, and this, it is probable, will mean that the rigid division now existing in theory between medicine and surgery will be broken down. One of our most distinguished physicians has protested against the compulsory application of this division as it exists at the present day, and it may be confidently anticipated that various mechanical methods of treatment will be practised by the physicians as freely as by the surgeons. In the second place it may be suggested that the progress of medicine has been a progress of prophylaxis, and that the treatment of the individual patient can show no such corresponding movement. And this comment must, at least in some measure, be admitted to be appropriate. Yet it is not the whole truth. To take but a single instance—namely, the treatment of diphtheria—it is common knowledge that the case mortality of this disease has, by the application of serum-therapeutics, been reduced by about 50 per cent. The use of thyroid extract in the treatment of myxœdema is yet another therapeutic triumph of the first moment. And, apart from these and other successes, it has to be recognised that the diagnosis of various diseases has been placed on a stable foundation; and accurate diagnosis is certainly the first step towards efficient treatment. Altogether medicine, as distinct from surgery, has reason both to be proud of its achievement and confident of its future.

